# Towards a classification of stem cells

**DOI:** 10.7554/eLife.46563

**Published:** 2019-03-13

**Authors:** Lucie Laplane, Eric Solary

**Affiliations:** 1 Institut d’Histoire et Philosophie des Sciences et des TechniquesUniversité Paris I Panthéon-SorbonneParisFrance; 2 Département d’HématologieInstitut de Cancérologie Gustave RoussyVillejuifFrance; 3 Faculté de MédecineUniversité Paris-SudLe Kremlin-BicêtreFrance

**Keywords:** Stemness, philosophy of science, philosophy of biology, cell fate, cancer stem cells, stem cells

## Abstract

The characteristic properties of stem cells – notably their ability to self-renew and to differentiate – have meant that they have traditionally been viewed as distinct from most other types of cells. However, recent research has blurred the line between stem cells and other cells by showing that the former display a range of behaviors in different tissues and at different stages of development. Here, we use the tools of metaphysics to describe a classification scheme for stem cells, and to highlight what their inherent diversity means for cancer treatment.

In certain multicellular organisms, stem cells can serve as reservoir of cells to produce, maintain, repair or even regenerate many tissues. Similarly, in cancer, a distinct fraction of cells, called cancer stem cells, may fuel the entire tumor as the disease emerges and progresses (Batlle and Clevers, 2017). How to define, isolate or characterize both healthy and cancer stem cells is a subject of much debate. Here, we provide a philosophical analysis of 'stemness' – the defining property of stem cells – arguing that this approach may shed new light on the nature of normal and malignant stem cells. We show that, depending on the circumstances, stemness may belong to one out of four distinct properties and discuss how this may influence therapeutic strategies in the oncology field.

## One size does not fit all

Stem cells have been described as a discrete population of cells sitting at the apex of a hierarchy of irreversible cell differentiation. This view has had an impact on the way in which stem cell research is performed by, for example, encouraging the idea that specific markers may help to distinguish and sort stem cells. However, researchers discovered that certain tissues showed much more plasticity in cell fate than anticipated. For example, it appeared that intestinal cells could replace the stem cells tasked with renewing the lining of the bowel (Tetteh et al., 2016). Such flexibility suggested that stemness might not be restricted to a pre-defined population of cells and prompted some biologists to question what stem cells really were. 

Traditional views of stem cells arose from studies of the hematopoietic tissue in the bone marrow, where blood cells originate in adults. However, in the late 1970s, Ray Schofield suggested that stemness actually relies on the interaction of hematopoietic stem cells (the cells that give rise to other blood cells) with the microenvironment in which they reside (Schofield, 1978). Although long denied, the importance of this 'niche' is now increasingly accepted: hematopoietic stem cells cannot be understood out of their context, which may account for the difficulties in maintaining them in culture.

Finally, new technologies, such as lineage tracing, have further questioned the boundaries of the stem cell category, as studies have shown that not every individual hematopoietic stem cell appears to be multipotent (discussed in Haas et al., 2018). These analyses highlighted that non-hematopoietic stem cells, called multipotent progenitors, could unexpectedly maintain the production of hematopoietic cells over an extended period of time (Sun et al., 2014). These are only a few of the examples that illustrate the potential diversity of these cells, which has resulted in two opposite views of what a stem cell is: it can either be an entity, a discrete population of cells with stable properties, or it can be a cell state, a property that is acquired in a specific context (e.g. Clevers and Watt, 2018; Zipori, 2004).

## Some order in the stemness mess

Philosophy, and more specifically metaphysics, has a long tradition of characterizing and distinguishing different types of properties for the objects or systems around us. We recently used this tradition to characterize stemness and our analysis led us to the conclusion that four types of properties may exist under the guise of stemness (Laplane, 2016; Laplane and Solary, 2017).

Stemness can be a 'categorical' property, that is, an intrinsic feature that is independent of any interaction with surrounding entities, such as the atomic mass of an element. It was initially assumed that all stem cells belong to this class, but, as discussed below, it might only apply to certain cancers.

Researchers discovered that certain tissues showed much more plasticity in cell fate than anticipated

Alternatively, stemness can be a 'dispositional' property, which is also an intrinsic feature, but one that only manifests upon interaction with external stimuli. For example, a fragile item only breaks on impact. Current knowledge suggests that healthy hematopoietic stem cells fall under this category. Although stemness is hardwired in these cells, it tightly depends on the bone marrow niche. In a malignant context, however, hematopoietic progenitor cells can gain stemness as they transform into malignant cells, questioning the maintenance of stemness as a dispositional property.

Stemness can also be a 'relational' property that relies on the interaction between entities. For example, body weight depends on gravity, and differs on the Earth and the Moon. Unlike the dispositional property, a relational property is not hardwired in a predetermined pool of cells, such as germline stem cells (which give rise to egg and sperm cells). When these stem cells are removed in fruit flies and mice, they are replaced by differentiated cells that migrate back to the stem cell niche, where they reacquire stemness (e.g. Brawley and Matunis, 2004). Stemness could also be a relational property in some cancers: recent research in a mouse model of colon cancer has revealed that after cancer stem cells had been eliminated, some remaining cancer cells regained stemness in the primary tumor but not in liver metastases, suggesting that a specific niche is required for the acquisition of stemness (de Sousa e Melo et al., 2017).

Finally, stemness can be a 'systemic' property, defined as an extrinsic characteristic that is provided and maintained by the system. For example, in the Matrix movie, every time the main antagonist Agent Smith is killed, the system transforms any human to incarnate the agent. Likewise, stemness is a systemic property when non-stem cells acquire stemness features in the absence of a specific environment, which may be the case in cancer. For example, some differentiated cells from breast cancer cell lines can become cancer stem cells again when cultured in vitro (Gupta et al., 2011). This suggests that stemness can be reacquired without a specific niche, with regulation taking place at the system level – here, the cancer cell population.

Stemness therefore encompasses distinct properties, depending on the tissue and context. Which category a stem cell population belongs to depends on two questions that can be addressed experimentally: first, can stemness be acquired by non-stem cells of that tissue? And second, is the niche mandatory for the acquisition of stemness, the expression of stemness, or both?

## Stemness in cancer

In oncology, the cancer stem cell model presupposes that cancers maintain an organization similar to that of healthy tissues, with a pool of malignant stem cells acting as the main reservoir for the production of every other cell of the tumor. These cells may be generated by the transformation of typical stem cells in the tissue of origin. The relationship between the rate of stem cell division and the risk of malignant transformation in a given tissue has led to much controversy in recent years (Tomasetti et al., 2017; Tomasetti and Vogelstein, 2015). Alternatively, cancer stem cells may emerge from non-stem cells endowed with stemness abilities thanks to genetic and epigenetic alterations. Similarities and dissimilarities between normal stem cells and cancer stem cells open three questions.

Stemness encompasses distinct properties, depending on the tissue and context

Firstly, is stemness the same in cancers and their non-pathological counterparts? Some data suggest that transformation can lead to, or rely on, a transition between two types of stemness. For instance, in some blood cancers known as myeloproliferative neoplasms, hematopoietic cells harboring the *JAK2* V617F mutation (which can be found in the majority of patients) secrete a molecule that damages the hematopoietic niche (Arranz et al., 2014). Only healthy, but not malignant stem cells, are affected by the degradation of their environment, which suggests that upon transformation into leukemic stem cells, normal stem cells become independent to the niche. This moves stemness from a dispositional property in healthy hematopoietic stem cells to a categorical property in leukemic stem cells.

Secondly, does stemness remain the same type of property throughout disease progression? The *JAK2* V617F mutation also contributes to shifting stemness from a dispositional to a categorical property by disrupting the regulation of hematopoietic stem cells by their environment (e.g., Staerk and Constantinescu, 2012). In other myeloid malignancies, oncogenic events such as *KRAS* mutations can lead to similar dysregulations. These mutations can occur either very early in leukemic transformation, or later during disease progression (Deininger et al., 2017). This suggests that stemness transitioning from a dispositional to a categorical property could also happen during the clonal evolution of the disease.

Lastly, is stemness the same type of property in all types of cancers of a particular tissue? In mouse models of squamous cell carcinoma, a type of skin cancer, cells gain migratory and invasive properties through a mechanism known as a epithelial-to-mesenchymal transition. This process can occur either frequently or rarely, depending on the chromatin state of the cell of origin (Latil et al., 2017). As this transition can result in stemness acquisition, squamous cell carcinomas may fall into two categories. In the absence of transition, stemness would best described as a dispositional property: intrinsic to the cells, but only revealed in the right context. However, in the presence of frequent transitions, stemness would become a relational property – extrinsic and conditional on epithelial-to-mesenchymal transitions.

Taken together, cancer occurrence and progression could be associated with changes in the nature of stemness. It remains to be seen whether these changes are a consequence of disease progression or one of its requirements.

## How stemness categorization may drive therapeutic choices

How is this philosophical characterization of stemness useful for science and medicine? We suggest that in oncology, the four types of stemness properties require different therapeutic strategies. Current treatment approaches target either malignant cells (with chemotherapy, ionizing radiations and targeted therapies), or surrounding cells (with immunotherapy or anti-angiogenic therapies). If these treatments fail to eliminate all the cancer stem cells, a relapse may occur. Choosing to target either cancer stem cells, their niche, or both, relies on implicit presuppositions regarding stemness.

Targeting cancer stem cells depends on the assumption that stemness is an intrinsic property (i.e., categorical or dispositional); otherwise, the malignant cells would regenerate new cancer stem cells after their elimination. Niche-targeting relies on the notion that stemness is a niche-dependent property (i.e., dispositional or relational). Accordingly, each type of stemness may determine the choice of a therapeutic approach:

- If stemness is a categorical property, only strategies that target cancer stem cells will be efficient.
- If stemness is a dispositional property, approaches that focus on both cancer stem cells and their niches may be used.
- If stemness is a relational property, only niche-targeting strategies will be potentially useful.
- If stemness is a systemic property, none of the therapeutic strategies aiming at eradicating cancer stem cells or their niche will be efficient, as the system (the cancer cell population) may instruct any tumor cell to become a cancer stem cell, without requiring any specific niche.

Changes in the nature of stemness may require therapeutic adaptation. It would thus be useful to monitor the impact of genetic and epigenetic alterations on the nature of stemness during the evolution of the disease. If changes in stemness are driving progression rather than resulting from it, therapeutic interventions that modulate stemness could also be beneficial.

## Perspective: is philosophy useful to science?

Stem cell biology is full of conceptual debates, and philosophers have contributed to these debates on a range of topics (reviewed in Fagan, 2013b), including the definition of stem cells and the entity/state debate (Fagan, 2013a; Laplane, 2016; Wilson et al., 2007; Leychkis et al., 2009). Focusing on the nature of stemness, we show how traditional tools of philosophy, such as the metaphysics of properties, can be applied to stem cell biology to shed light on the fundamental nature of stem cells. More than a simple descriptive characterization, the proposed classification draws practical consequences in guiding the choice of an anticancer treatment. Many questions remain unsolved, however. If stemness is not one but four different properties, are we right to use only one name? Are all the cells that we call stem cells, really stem cells? Do they share some underlying characteristics that justify their biological clustering, or is 'stem cell' just a convenient category to group cell types that are actually distinct?

While experimental biology will hardly answer these questions, phylogeny, on the other hand, could be helpful. For instance, mammals and insects have distinct types of eyes that have emerged independently throughout evolution as two different but effective solutions to a similar challenge. Similarly, a phylogenetic analysis of stemness could highlight whether the different types of stem cells occurred separately during evolution, as different solutions to the challenges of tissue maintenance and repair. In turn, this would deepen the metaphysics of stem cells by identifying whether our four stemness properties depict entities that are biologically independent . It is only by bridging disciplines such as experimental biology, medicine, phylogeny and philosophy that stem cells will be properly understood.

## Note 

This Feature Article is part of the Philosophy of Biology collection. 

## References

[bib1] Arranz L, Sánchez-Aguilera A, Martín-Pérez D, Isern J, Langa X, Tzankov A, Lundberg P, Muntión S, Tzeng YS, Lai DM, Schwaller J, Skoda RC, Méndez-Ferrer S (2014). Neuropathy of haematopoietic stem cell niche is essential for myeloproliferative neoplasms. Nature.

[bib2] Batlle E, Clevers H (2017). Cancer stem cells revisited. Nature Medicine.

[bib3] Brawley C, Matunis E (2004). Regeneration of male germline stem cells by spermatogonial dedifferentiation *in vivo*. Science.

[bib4] Clevers H, Watt FM (2018). Defining adult stem cells by function, not by phenotype. Annual Review of Biochemistry.

[bib5] de Sousa e Melo F, Kurtova AV, Harnoss JM, Kljavin N, Hoeck JD, Hung J, Anderson JE, Storm EE, Modrusan Z, Koeppen H, Dijkgraaf GJ, Piskol R, de Sauvage FJ (2017). A distinct role for Lgr5^+^ stem cells in primary and metastatic colon cancer. Nature.

[bib6] Deininger MWN, Tyner JW, Solary E (2017). Turning the tide in myelodysplastic/myeloproliferative neoplasms. Nature Reviews Cancer.

[bib7] Fagan MB (2013a). Philosophy of stem cell biology - an introduction. Philosophy Compass.

[bib8] Fagan MB (2013b). Philosophy of Stem Cell Biology: Knowledge in Flesh and Blood.

[bib9] Gupta PB, Fillmore CM, Jiang G, Shapira SD, Tao K, Kuperwasser C, Lander ES (2011). Stochastic state transitions give rise to phenotypic equilibrium in populations of cancer cells. Cell.

[bib10] Haas S, Trumpp A, Milsom MD (2018). Causes and consequences of hematopoietic stem cell heterogeneity. Cell Stem Cell.

[bib11] Laplane L (2016). Cancer Stem Cells: Philosophy and Therapies.

[bib12] Laplane L, Solary Éric (2017). Identité des cellules souches normales et cancéreuses. Médecine/Sciences.

[bib13] Latil M, Nassar D, Beck B, Boumahdi S, Wang L, Brisebarre A, Dubois C, Nkusi E, Lenglez S, Checinska A, Vercauteren Drubbel A, Devos M, Declercq W, Yi R, Blanpain C (2017). Cell-type-specific chromatin states differentially prime squamous cell carcinoma tumor-initiating cells for epithelial to mesenchymal transition. Cell Stem Cell.

[bib14] Leychkis Y, Munzer SR, Richardson JL (2009). What is stemness?. Studies in History and Philosophy of Science Part C: Studies in History and Philosophy of Biological and Biomedical Sciences.

[bib15] Schofield R (1978). The relationship between the spleen colony-forming cell and the haemopoietic stem cell. Blood Cells.

[bib16] Staerk J, Constantinescu SN (2012). The JAK-STAT pathway and hematopoietic stem cells from the JAK2 V617F perspective. JAK-STAT.

[bib17] Sun J, Ramos A, Chapman B, Johnnidis JB, Le L, Ho YJ, Klein A, Hofmann O, Camargo FD (2014). Clonal dynamics of native haematopoiesis. Nature.

[bib18] Tetteh PW, Basak O, Farin HF, Wiebrands K, Kretzschmar K, Begthel H, van den Born M, Korving J, de Sauvage F, van Es JH, van Oudenaarden A, Clevers H (2016). Replacement of lost Lgr5-positive stem cells through plasticity of their enterocyte-lineage daughters. Cell Stem Cell.

[bib19] Tomasetti C, Li L, Vogelstein B (2017). Stem cell divisions, somatic mutations, cancer etiology, and cancer prevention. Science.

[bib20] Tomasetti C, Vogelstein B (2015). Variation in cancer risk among tissues can be explained by the number of stem cell divisions. Science.

[bib21] Wilson RA, Barker MJ, Brigandt I (2007). When traditional essentialism fails: biological natural kinds. Philosophical Topics.

[bib22] Zipori D (2004). The nature of stem cells: state rather than entity. Nature Reviews Genetics.

